# A global high-density chromatin interaction network reveals functional long-range and trans-chromosomal relationships

**DOI:** 10.1186/s13059-022-02790-z

**Published:** 2022-11-09

**Authors:** Ruchi Lohia, Nathan Fox, Jesse Gillis

**Affiliations:** 1 Stanley Institute for Cognitive Genomics, Cold Spring Harbor Laboratory, Cold Spring Harbor, USA; 2 Department of Physiology and Donnelly Centre for Cellular and Biomolecular Research, University of Toronto, Toronto, Canada

**Keywords:** Hi-C, Meta-analysis, Coexpression, eQTL

## Abstract

**Background:**

Chromatin contacts are essential for gene-expression regulation; however, obtaining a high-resolution genome-wide chromatin contact map is still prohibitively expensive owing to large genome sizes and the quadratic scale of pairwise data. Chromosome conformation capture (3C)-based methods such as Hi-C have been extensively used to obtain chromatin contacts. However, since the sparsity of these maps increases with an increase in genomic distance between contacts, long-range or trans-chromatin contacts are especially challenging to sample.

**Results:**

Here, we create a high-density reference genome-wide chromatin contact map using a meta-analytic approach. We integrate 3600 human, 6700 mouse, and 500 fly Hi-C experiments to create species-specific meta-Hi-C chromatin contact maps with 304 billion, 193 billion, and 19 billion contacts in respective species. We validate that meta-Hi-C contact maps are uniquely powered to capture functional chromatin contacts in both cis and trans. We find that while individual dataset Hi-C networks are largely unable to predict any long-range coexpression (median 0.54 AUC), meta-Hi-C networks perform comparably in both cis and trans (0.65 AUC vs 0.64 AUC). Similarly, for long-range expression quantitative trait loci (eQTL), meta-Hi-C contacts outperform all individual Hi-C experiments, providing an improvement over the conventionally used linear genomic distance-based association. Assessing between species, we find patterns of chromatin contact conservation in both cis and trans and strong associations with coexpression even in species for which Hi-C data is lacking.

**Conclusions:**

We have generated an integrated chromatin interaction network which complements a large number of methodological and analytic approaches focused on improved specificity or interpretation. This high-depth “super-experiment” is surprisingly powerful in capturing long-range functional relationships of chromatin interactions, which are now able to predict coexpression, eQTLs, and cross-species relationships. The meta-Hi-C networks are available at https://labshare.cshl.edu/shares/gillislab/resource/HiC/.

**Supplementary Information:**

The online version contains supplementary material available at 10.1186/s13059-022-02790-z.

## Background

The physical associations generated by chromatin contacts are a critical factor to regulate and determine gene-expression patterns [1–4]. Functional chromatin contacts can form across a wide range of genomic distances within a chromosome (cis) or across chromosomes (trans). Although trans contacts are non-random [5] and there is evidence of trans-regulatory interactions [6, 7], studying the functional role of these interactions is difficult due to the high sparsity of available chromatin contact maps in trans.

**Fig. 1:**
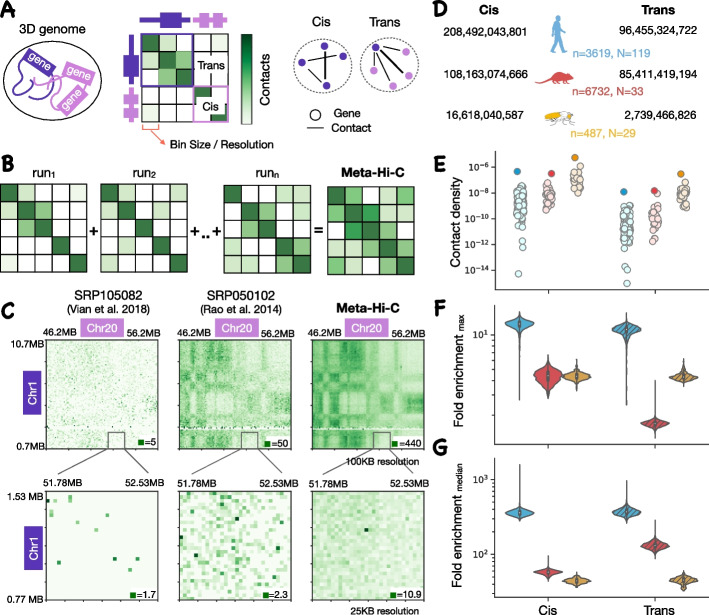
Creating the meta-Hi-C network. **A** Genes are co-localized in chromatin 3D structure through frequent chromatin contacts. Each chromosome is divided up into “bins” of a specific size referred to as resolution and the chromatin contact matrix represents the number of pair-ended reads spanning between a pair of bins. The contact matrix can be represented with networks where nodes are genes and edges are interaction frequencies. The cis and trans networks consist of intra-chromosome and inter-chromosome edges respectively. **B** Contact maps from individual Hi-C experiments are aggregated to create a meta-Hi-C contact map. **C** Visual comparison of our meta-Hi-C and two individual Hi-C contact matrices: Vian et al. [9] (a typical experiment) and Rao et al. [10] (densest experiment in trans) at 100-KB (top) and 25-KB resolutions (bottom). The maximum contact frequency is given at the bottom of each map. The high-intensity region observed in our meta-Hi-C contact matrix at 25-KB resolution belongs to gene UBEJ2 on Chr1 and ZFP64 on Chr20. Both these genes are also strongly coexpressed in our coexpression network with a coexpression strength of 0.98 on a genome-wide rank-standardized scale of 0 to 1. **D** Total number of contacts in cis and trans meta-Hi-C network of human, mouse, and fly. *n* and *N* are the numbers of runs and projects aggregated in each species respectively. **E** Contact density (total contacts/number of 1-bp bins) across individual projects in cis and trans for each species. Individual points are individual experiments and the darker shades of points are the values for the meta-Hi-C network. Distribution of fold enrichment of contacts among genes in the meta-Hi-C network relative to the maximum (**F**) or median (**G**) number of contacts for that gene among individual network in cis and trans. The total number of genes in **F** and **G** are 23,465, 20,672, and 9636 in human, mouse and fly respectively

Obtaining high-density chromatin contact maps at all genomic distances and in trans is not yet feasible with most existing maps being essentially probabilistic in nature, capturing some fraction of likely present contacts in a distance-dependent manner. Genome-wide contact maps can be obtained using chromosome conformation capture (3C)-based technologies such as Hi-C [8]. From Hi-C experiments, an *nxn* chromatin contact matrix is generated where the genome is divided into* n* equally sized bins and contact frequency between each bin-pair is obtained by summing the pair-ended reads spanning between a pair of bins (Fig. 1A). The bin size is also referred to as “resolution” and is dependent on the sequencing depth of the experiment — at low sequencing depth, the matrix is generated using large bin sizes (low resolution) to reduce the sparsity of the matrix. The commonly used sizes of these bins can range from 1 KB to 1 MB. However, due to large genome sizes and the quadratic scale of pairwise data, obtaining these chromatin contact matrices at high resolution would require prohibitively expensive sequencing at even 1X depth in the pairwise space. Capturing long-range and trans chromatin contacts is made more difficult since the frequency of contacts decreases with an increase in genomic distance between contacting loci in cis [8]. And in trans, the contacts are at least 2 orders of magnitude less frequent [5] while also having a larger search space than cis.

To overcome the sequencing-depth barrier, targeted 3C-based techniques such as ChiA-PET [11] and Capture-Hi-C [12] are widely used to obtain high-resolution contact maps for specific proteins or selected loci respectively. Alternatively, several in silico methods have taken the advantage of existing limited-resolution contact maps to either generate higher resolution maps using machine learning approaches [13–16] and/or detect statistically significant interactions by background fitting [10, 17]. However, with a few exceptions [18, 19], most of the available methods are only tested to enhance cis interactions because longer range interactions are essentially unavailable within any given data set.

In this work, we propose a meta-analysis approach where we leverage several hundreds of available chromatin contact matrix generated from Hi-C-based experiments to create a dense genome-wide chromatin contact matrix for three species: human, mouse, and fly. We show that these meta-Hi-C chromatin contact matrices are valuable for capturing long-range and trans-chromosomal interactions. We evaluated the effectiveness of contact maps using three criteria; chromatin contact matrix was used to predict (1) gene-expression profiles, (2) target genes for eQTLs, and (3) conservation across pairs of species (human-mouse, human-fly, and mouse-fly). Our reference networks complement a very diverse array of efforts in genomics, from those focused on more targeted experiments in Hi-C which now have an overall “null” with which to compare individual results, to genome interpretation methods, whether interpreting variants, expression patterning, or regulatory sequence.

## Results

### Meta-Hi-C network predicts coexpression at greater resolution and scale than individual networks

In brief, for building the meta-Hi-C matrix, we uniformly processed 3619, 6732, and 487 Hi-C runs for human, mouse, and fly respectively. The runs were obtained after querying sequence read archive (SRA) with field limitations of given species and Hi-C as experiment strategy. A genome-wide chromatin contact matrix at several resolutions (1KB, 5KB, 10KB, 25KB, 40KB, 100KB, 250KB, and 500KB) was created for each run after mapping the reads to the same reference genome for each species. Within SRA, all the runs belonging to a study are grouped together as a project. A project can consist of multiple runs, which can include biological or technical replicates across multiple tissues or cell types. Chromatin contact matrices within a project were aggregated to create a project-level Hi-C matrix. There were 119, 33, and 29 projects for human, mouse, and fly respectively (Fig. 1D). The meta-Hi-C matrix for each species was created after further aggregating all project-level Hi-C matrix within a species (Fig. 1B). For subsequent analysis, the genome-wide Hi-C matrix was mapped to genes to create Hi-C networks where nodes are genes and edges are the interaction frequency between genes (Fig. 1A). To determine contact frequency between each gene pair we use the maximum contact frequency between each bin in which genes reside. The genome-wide Hi-C networks were divided into cis and trans depending on if the edge connects two genes in the same chromosome or different chromosomes respectively. Figure 1D–G highlights the comprehensively greater depth of the meta-Hi-C network. To validate the predictive power of the meta-Hi-C network, we benchmarked it against Hi-C networks inferred from individual projects for each species.

**Fig. 2:**
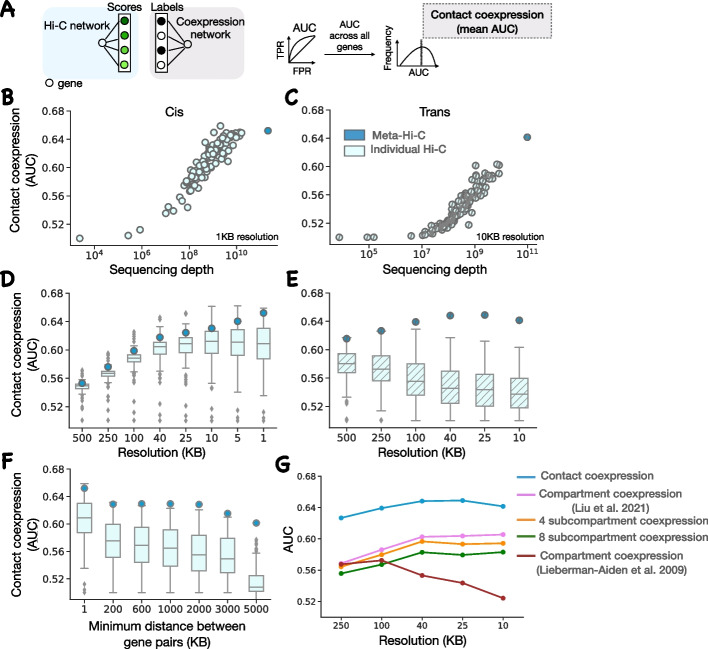
Meta-Hi-C network benchmarking. **A** Contact coexpression metric schematic. Circles represent genes and lines represent edges of that gene in respective networks. For each target gene, we use its ranked edges in the Hi-C network to predict the top 1% of its edges in the coexpression network. We perform this task for every gene and then report contact coexpression as the average AUC across all genes. **B** Contact coexpression for the individual and meta-Hi-C network in cis as a function of sequencing depth at 1-KB resolution **C** Same as (**B**) but in trans and at 10-KB resolution. **D** The boxplot shows the distribution of contact coexpression for each project at various resolutions in cis. Circles represent the performance of the cis meta-Hi-C network. **E** Same as (**D**) but using trans networks. **F** The boxplot shows the distribution of contact coexpression in cis at 1-KB resolution for each project at various distance thresholds. **G** Comparison of contact coexpression score of the meta-Hi-C network and compartment coexpression score at various resolutions. We called compartments in each individual network and then aggregated those calls, capturing the probability of sharing a compartment across data. The compartment coexpression metric captures the ability of aggregated compartment preference to predict coexpression. Subcompartment coexpression is defined analogously to compartment coexpression. Compartments were called using either the Liu et al. [23] method or the Liberman-Aiden et al. [8] method

As our first performance test, we assessed the tendency for spatially co-localized genes to be coexpressed [20, 21], using previously derived shared patterns of expression in independent data [22]. The underlying hypothesis is that spatial proximity may be a useful way to organize regulatory relationships, as in the case of linear sequence, thus yielding shared spatial relationships for genes that are coexpressed. Thus, while perfect performance at predicting coexpression is not expected, the genome-wide scale of the assessment makes it useful for assessing cis and trans effects. For each gene, we measure the ability of interaction frequency to predict the gene’s top 1% coexpression partners. We call this measure “contact coexpression” (Fig. 2A) and is expressed as an AUC (area under the ROC curve) with possible values ranging between 0 and 1. A score of 1 indicates that interaction frequency perfectly predicts coexpression; 0.5 indicates no relationship.

We evaluated the contact coexpression as a function of the sequencing depth of the Hi-C network in cis (Fig. 2B) and trans (Fig. 2C) for all individual Hi-C networks and meta-Hi-C network in human. We find that performance is linearly dependent on the log of sequencing depth and meta-analysis provides additional coverage. We find that in cis the best powered individual experiments are close to the saturation depth that maximizes performance (Fig. 2B), although performing substantially worse in trans. In trans, the meta-Hi-C network acts like a “super-experiment”, where the additional coverage fully converts into substantial additional performance (Fig. 2C). We found similar results for mouse (Additional file 1: Fig. S1C and S1D) and fly (Additional file 1: Fig. S2C and S2D). Although contact coexpression scores in cis and trans are similar (0.63 AUC cis vs 0.64 AUC trans at 10KB resolution), the search space in trans is at least three times larger when compared to cis, making a direct comparison of the aggregate “strength” of cis and trans relationships non-trivial.

The resolution of the Hi-C matrix is an important parameter for obtaining gene networks, and hence, we evaluated the contact coexpression of individual Hi-C and meta-Hi-C networks at several resolutions. We find that for the individual networks performance increases with an increase in resolution, plateaus, and then slightly falls off in cis (Fig. 2D). In essence, improved resolution is useful in cis because the coverage is adequate for it to provide a useful signal until the very finest resolution where most experiments begin to decline, although the meta-Hi-C network continues to slightly increase, as might be expected. In contrast, in trans (Fig. 2E), the performance monotonically falls with an increase in resolution for individual experiments. However, the trans pattern for meta-Hi-C networks strongly resembles that of individual experiments in cis, increasing and then plateauing with improvements in resolution. This suggests unlike individual networks, meta-Hi-C networks are dense enough to be analyzed at high resolutions even in trans. We found similar results for mouse (Additional file 1: Fig. S1A and S1B) and fly (Additional file 1: Fig. S2A and S2B).

The Hi-C matrix in cis has high density of contacts at bins near the diagonal and the contact density decreases exponentially as the distance between the bins increases so that even Hi-C networks with higher contact density on average will be highly sparse at distant bins. This makes it difficult to capture functional contacts between distant gene pairs from a Hi-C matrix. Hence, we evaluated the contact coexpression of individual Hi-C networks and meta-Hi-C networks at various linear distance thresholds in cis. We find that for long-range contacts (minimum distance between gene pairs> 600 KB) the additional sequencing depth of meta-Hi-C networks when compared to individual Hi-C networks fully converts into additional performance (Fig. 2F). However, for both individual networks and meta-Hi-C network, the performance decreases in the absence of short-range contacts. This could be due to a higher number of short-range regulatory interactions or due to the similarity of the chromatin environment for nearby genes.

Chromatin can be folded into structural patterns at different length scales [24]. At a large genomic scale, the genome is spatially segregated into two compartments [8] and further up to 8 subcompartments [23]. At a shorter genomic scale (< 1 MB), chromosomes fold into topologically associated domains (TADs). Since the interaction between genes is partly constrained to occur within the same structural units (compartment/subcompartment/TADs), we sought to determine if the contact coexpression performance of the meta-Hi-C network can be explained with these genomic structures. In each individual network, we identified the compartment of each gene and then binarized the compartment preference for each gene pair: so each experiment becomes a binary network of gene pairs found in the same compartment (1) or a different compartment (0). The binarized individual networks are then aggregated, capturing the probability of sharing a compartment across all data. For each gene, we measure the ability of aggregated compartment frequency to predict the gene’s top 1% coexpression partners. We call this measure “compartment coexpression” and it is expressed as an AUC with possible values ranging between 0 and 1. A score of 1 indicates that the same compartment frequency perfectly predicts coexpression; 0.5 indicates no relationship. We also defined “subcompartment coexpression” and “TAD coexpression” analogously to compartment coexpression.

We compared TAD coexpression (defined only in cis), compartment coexpression, and subcompartment coexpression with meta-Hi-C contact coexpression at several resolutions. We used two different methods for calling compartments: an older PCA-based method Liberman-Aiden et al. [8] and a comparatively recent method Calder [23]. In cis, we find that compartment and subcompartment coexpression is comparable to or better than contact coexpression while TAD coexpression is lower than compartment at up to 10-KB resolution (Additional file 1: Fig. S3). TADs are often considered functional genomic units and genes within the same TADs tend to be coexpressed [25]. However, unlike compartment and contact coexpression, TAD coexpression does not capture long-range interactions (average TAD size is smaller than 1 MB). This likely explains the non-random yet low performance of TAD coexpression (AUC 0.55). We also evaluated the conservation of TADs and boundaries across all individual Hi-C matrices (Additional file 1: Fig. S4). The number of TADs conserved across experiments decreases relatively rapidly and we did not find any TAD which was conserved across all the experiments. In trans, we find that compartment coexpression and subcompartment coexpression performances are lower than contact coexpression performance, suggesting other trans interactions contribute (Fig. 2G).

**Fig. 3:**
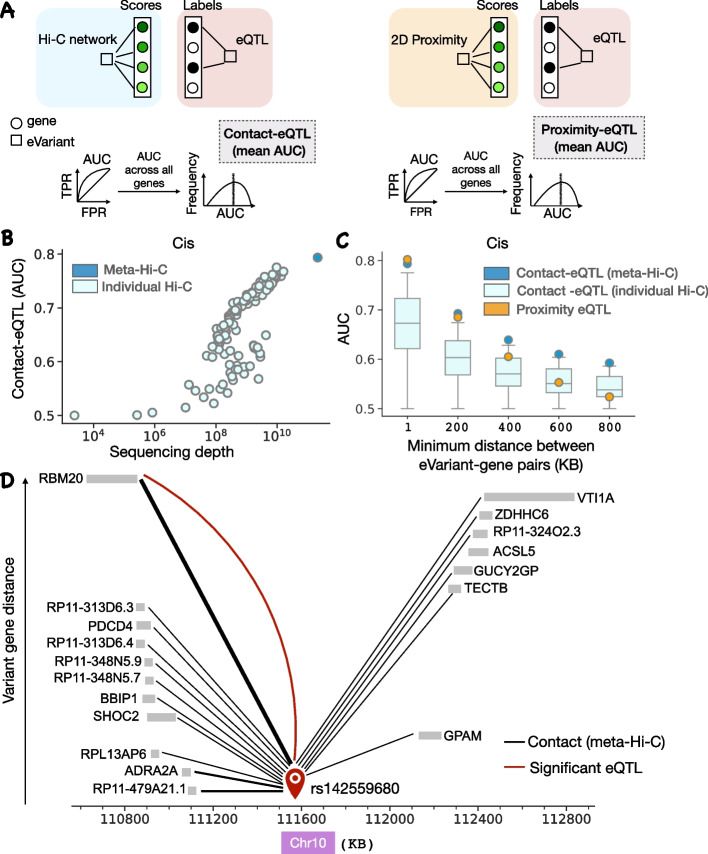
Meta-Hi-C network captures long-range eQTLs. **A** Contact-eQTL and proximity-eQTL metric schematic. For contact-eQTL and proximity-eQTL, for each variant, the edges are ranked by contact frequency or inverse of the genomic distance from the gene respectively. The labels are obtained from eQTL associations (Section Methods). We perform this task for every variant and then report the average AUC across all variants. **B** Contact-eQTL for individual Hi-C network and meta-Hi-C network at 1-KB resolution as a function of sequencing depth. **C** The boxplot shows the distribution of contact-eQTL for each project at various minimum distance thresholds. Circles represent the contact-eQTL of the meta-Hi-C network and proximity-eQTL. **D** An example comparing contact-eQTL with proximity-eQTL. All genes associated with eVariant rs142559680 along with their sequence position on the *X* axis. The vertical separation between gene and variant increases as the distance between the variant and gene TSS increases. The thickness of the variant gene edge increases with an increase in the contact frequency in the meta-Hi-C network. Although gene RP11-479A21.1 is the closest to the variant, gene RBM20 has the strongest contact with the variant and is also the only significantly associated gene with the variant

### Meta-Hi-C network effectively captures more eQTL interactions

For our second performance assessment, we tested the hypothesis that genetic variants (eVariant) regulate gene expression of the target gene (eGene) via physical contact [26, 27]. The set of eQTLs was obtained from GTEx (Section Methods). For each eVariant, the interaction frequency with all genes falling in unique contact map bins at 1-KB resolution was used to predict the eGene (Fig. 3A). This is termed “contact-eQTL” and is expressed as an AUC with possible values ranging between 0 and 1, with 1 and 0 meaning that the eVariant and target eGenes have the highest and lowest interaction frequency respectively when compared to all eVariant and non-eGene interactions. Similar to the previous benchmarking test, we find that performance is linearly dependent on the log of sequencing depth and meta-analysis provides additional coverage; the meta-Hi-C network has higher performance when compared to any of the individual Hi-C networks (Fig. 3B). This emphasizes the significance of dense Hi-C networks in identifying eQTLs.

We further evaluated the ability of meta-Hi-C networks to predict target genes for variants by comparing their performance with a linear genomic distance-based predictor, the current standard approach. The distance between the variant and gene transcription start site (TSS) remains almost the only metric widely used to annotate target genes for variants [28]. For each eVariant, the inverse of linear distance (1/TSS) with all genes is ranked and then used to predict the eGene (Fig. 3A). This is termed “proximity-eQTL” and is expressed as an AUC with possible values ranging between 0 and 1, with 1 and 0 meaning that eGenes are the closest and farthest from the eVariant respectively. We compared contact-eQTL of individual Hi-C networks and meta-Hi-C networks at various linear distance thresholds (Fig. 3C). We reassuringly find that the meta-Hi-C network outperforms individual Hi-C networks at all distance thresholds. Interestingly, we find that contact-eQTL (meta-Hi-C) outperforms proximity-eQTL for long range eQTLs (minimum distance between the eVariant-gene pairs > 200KB). Furthermore, the performance for both contact-eQTL and proximity-eQTL decreases in the absence of short-range contacts. This is in agreement with our previous observation where we find that contact coexpression decreases in the absence of short-range contacts.

### Trans-chromosomal chromatin contacts show evolutionary conservation

Having established that meta-Hi-C networks are well powered to capture meaningful contacts, we now use them to study the conservation of genomic contacts between species. Since chromatin contacts regulate gene expression, it is reasonable to expect some conservation of these contacts across species even in the context of large scale genomic alteration and, in the reverse, divergence in contacts across species can help explain regulatory evolution [29, 30]. We evaluated the conservation of contacts across species in three different ways; we compare the contact coexpression scores for ortholog genes in each species pair, and we use the Hi-C network of one species to predict either Hi-C network (“contact conservation”) or coexpression network in another species.

**Fig. 4:**
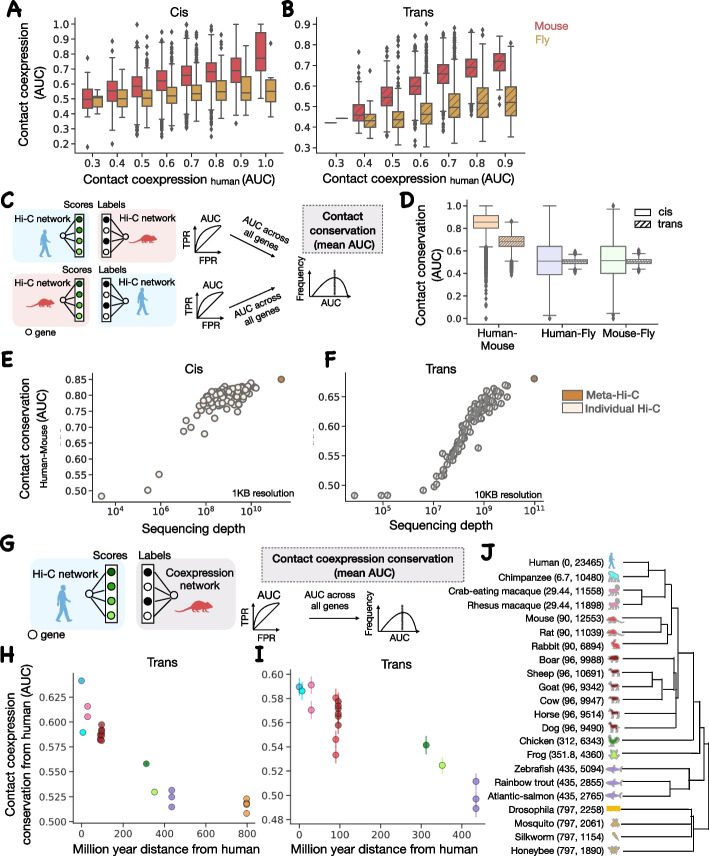
Chromatin contacts are conserved across species in both cis and trans. Contact coexpression in cis (**A**) and trans (**B**) for 1:1 orthologs in human-mouse and human-fly. **C** Contact conservation schematic. For each gene, ranked edges in human Hi-C network are used to predict the top 10% of mouse Hi-C network edges. This task is repeated for each gene and in both directions and the average AUC is reported as human-mouse contact conservation. **D** The distribution of contact conservation score across genes in each direction using the meta-Hi-C network for various pairs of species. human-mouse contact conservation for individual Hi-C network and meta-Hi-C network as a function of sequencing depth in cis (**E**) and trans (**F**). **G** Contact coexpression conservation schematic. For each gene, ranked edges in the human Hi-C network are used to predict the top 1% of mouse coexpression network edges. This task is repeated for each gene and we report the average AUC as mouse contact coexpression conservation with human. **H** Contact coexpression conservation with human for several species. **I** Same as (**H**) but only using the same set of 429 ortholog genes across species. The error bars represent a 68% confidence interval. In cis, the Hi-C network is analyzed at 1-KB resolution, and in trans, the Hi-C network at 10-KB resolution is used for human and mouse and 1-KB resolution for fly. **J** The dendrogram shows phylogenetic relationships between species used for contact coexpression conservation analysis. Million year distance from human and number of 1:1 orthologs with human is listed in the parenthesis

Before directly comparing the contact map across species, we first compared the contact coexpression scores for 1:1 orthologous genes across species. We find a strong linear relationship between human and mouse scores and a somewhat weaker relationship between human and fly scores in both cis (Fig. 4A) and trans (Fig. 4B). This suggests that if a gene is spatially co-regulated in one species, it is likely to be spatially co-regulated across other species.

We next characterized the degree to which gene contacts are conserved by directly comparing the meta-Hi-C network across species. Each gene’s shared neighborhood is defined by ranking all edges in the chromatin contact network and then using it to predict the gene’s top 10% of edges in another species. We call this “contact conservation” and again treat it as a prediction task with 1 meaning perfect contact conservation, 0.5 consistent with random reordering of neighborhoods, and 0 meaning that contacting partners have reversed (Fig. 4C). For the trans conservation score, only the trans gene pairs in both species are used, similarly for cis analysis. As expected, we find that the contact conservation is higher for human-mouse (AUC> 0.8) when compared with human-fly (AUC 0.5) or mouse-fly (AUC 0.51) (Fig. 4D) in both cis and trans.

We also re-validated the power of the meta-Hi-C network: we compared the contact conservation scores for individual and meta-Hi-C networks at the highest available resolution. We reassuringly find that the meta-Hi-C network outperforms individual projects in both cis and trans (Fig. 4E, F). This again suggests that the meta-Hi-C network is efficient in capturing chromatin contacts when compared to individual networks. Although the conservation of cis chromatin structure across species is not surprising and is evident in the presence of syntenic regions between species, the conservation of trans-chromatin contacts is noteworthy. It suggests that the trans-chromatin structure is likely selected for preservation to maintain function.

We further investigated the evolution of trans chromatin contacts in human by comparing the degree to which the human contacts can predict coexpression across several species. This method allowed us to extend our analysis to species for which the meta-Hi-C network is not available. Each gene’s neighborhood is defined by ranking all edges in the chromatin contact network of one species and then used to predict the gene’s top 1% of coexpressed gene pairs in another species. We call this “contact coexpression conservation” and calculate the AUC as above (Fig. 4G). When contact coexpression conservation is plotted along with the phylogenetic distance across species, we find that the performance decreases with an increase in phylogenetic distance using trans meta-Hi-C networks (Fig. 4H). This suggests that the contacts diverge as the species pair becomes distant across evolution. As expected, we also find that the strongest coexpressed genes have the most contact coexpression conservation (Additional file 1: Fig. S5). The number of 1:1 orthologs also decreases with an increase in the phylogenetic distance (Fig. 4J), and it seemed possible that our observation was dominated by the number of ortholog pairs between species. To eliminate this possibility, we redid our analysis but using only the same set of ortholog genes (429 genes) in each species and our result persisted (Fig. 4I). Species more than 100 million years of distance (mya) from humans have stronger divergence in contacts when compared to species within the Mammalia Class. The species included in Fig. 4I were limited to the Chordata phylum to ensure a reasonable number of genes in the analysis.

### Data availability and online tool

In order to facilitate the broad adoption of meta-Hi-C by the community, we have made data available via an online tool (https://gillisweb.cshl.edu/HiC/). Contact data can be obtained in two ways: (a) network download: direct download of the desired resolution and species meta-Hi-C contact matrix in cis or trans available at https://labshare.cshl.edu/shares/gillislab/resource/HiC in HiCMatrix format (https://github.com/deeptools/HiCMatrix) and (b) gene vector download: contact frequency with every genomic loci at the chosen resolution and for any desired gene found in the respective species. The downloaded file is in a bed file format which can be uploaded to the UCSC genome browser for further analysis as desired.

## Discussion

In this work, we created a high-depth, genome-wide chromatin contact map using a meta-analytic approach, validated it, and further used it to reveal chromatin structure to function relationships. We find that for the three species analyzed in this study (human, mouse, and fly), chromatin contacts strongly predicted the coexpression of genes. We also show that chromatin contacts are better than linear proximity for predicting eQTLs when high-resolution chromatin contact data is available. Our results persist even when only long-range chromatin contacts are analyzed. Additionally, we find that trans-chromosomal contacts show evidence of conservation between human and mouse. In contrast, we do not find any conservation between these mammalian networks and fly (human-fly AUC 0.50, mouse-fly AUC 0.51). This is striking in its nearly exact correspondence with the null, but the very large amount of data combined with the dramatically different genomes appears to leave no average signal when analyzed across homologs. It remains plausible that a model trained specifically to account for the broad cross-species differences would find subtler biological overlaps our direct assessments miss.

Meta-Hi-C networks are an effective means for capturing otherwise hard to characterize long-range interactions providing potentially uniquely important practical applications. One important application for a wide area of genomics is their ability to prioritize distant target genes for variants. We expect these networks to be powerful training data for future machine learning attempts to predict chromosomal contacts, an important area of ongoing research [13–16]. Additionally, meta-Hi-C networks can be used with other cell-type-specific ‘omics datasets such as ChIP-Seq to reveal cell-type-specific enhancer-promoter contacts. Previously, Nasser et al. [31, 32] used averaged Hi-C data across 10 cell types in their ABC model to accurately make cell-type-specific enhancer-gene predictions. Thus, the continuing evolution of methods with improved specificity is likely to complement our better-powered but less condition-specific meta-analytic approach.

Within the Hi-C analysis, and even outside of it, aggregation of data is well appreciated to be a useful strategy. Reproducible biological replicates within the same study are often combined to increase the density of Hi-C data thereby capturing more interactions [10, 33]. Our approach can be thought of as the most extreme version of this idea, combining experiments as broadly as possible to capture statistical relationships that are common. This is most useful if the depth is a major limitation, as in trans contacts, as it comes with the cost of a loss of condition-specificity. Thus, the route forward for the field as a whole will doubtless involve both improved specificity, integration, and interpretive methods.

In summary, our study sheds new light on the functional role of long-range and trans-chromosomal contacts and provides a critical resource for use by a wide range of genomics research.

## Conclusions

In this study, we leveraged the hundreds of available Hi-C maps by aggregating them to build high-depth meta-Hi-C maps for human, mouse, and fly. These maps act like a “super-experiment” where additional depth leads to surprising power in capturing long-range functional relationships. These maps are able to predict coexpression, eQTLs, and cross-species relationships. The availability of the meta-Hi-C maps complements the ongoing efforts in identifying functional chromatin contacts and provides significant evidence for the functionality of trans chromatin contacts. The meta-Hi-C networks are available for download via an online tool at https://gillisweb.cshl.edu/HiC/.

## Methods

### Hi-C data sources

The Hi-C data for each species were obtained from SRA search (https://www.ncbi.nlm.nih.gov/sra/) with the field limitations of “Organism”: [“Homo sapiens,” “Mus musculus,” “Drosophila melanogaster”], “Strategy”: “hi c.” We found 3913, 8431, and 502 runs for human, mouse, and fly respectively. We also added 268, 17, and 25 runs manually that were labeled OTHER in SRA, but were deemed to be valid Hi-C data based on publication details. After manual additions, filtering out runs without available restriction enzyme information, and excluding runs that failed to process, we had 3621, 6733, and 487 samples for human, mouse, and fly.

SRA projects processed for human are SRP050102 [10], SRP012412 [34], SRP152979 [35], SRP152879 [36], SRP118999 [37], SRP154953 [38], SRP199098 [39], SRP234115 [40], SRP094854, SRP149906 [36], SRP165933 [41], SRP212226 [14], SRP117084 [42], SRP218691, SRP233368 [43], SRP106040 [44], SRP125488, SRP250432, SRP141473 [45], ERP107279, SRP224133 [46], SRP184300, SRP168606 [29], SRP216194 [47], SRP178527 [48], SRP173234 [49], SRP239849 [50], SRP106379, SRP162098 [51], SRP120957, DRP005280, SRP150259 [52], SRP170743, SRP131003 [53], SRP158113, SRP186190, SRP212073 [54], SRP133031 [55], SRP135798, SRP131871 [56], SRP158276 [57], SRP114754, SRP267107 [58], SRP227918 [59], SRP271101 [60], SRP186277, SRP115913 [61], SRP157799 [62], SRP110964, SRP194362 [63], SRP151075 [64], SRP157894 [65], SRP160101, SRP157048 [66], SRP221518 [67], SRP225696 [68], ERP104251, SRP105082 [9], SRP223060 [69], SRP234897 [70], SRP250333 [71], SRP113633 [72], SRP186012 [73], SRP199225 [74], SRP107176 [75], SRP105181 [76], SRP066852, SRP095110, SRP162056, SRP201909 [77], SRP153415 [78], SRP127183 [79], ERP118600, SRP274139 [80], SRP115572 [81], SRP099610 [82], SRP108500 [83], SRP195614 [84], SRP235557 [85], SRP264796 [86], SRP197114, SRP132233 [87], SRP244334, SRP113478, SRP107148, SRP083971, SRP192392 [88], SRP145420 [89], SRP152361 [90], SRP141229, SRP261300, SRP154986 [91], SRP261299, SRP150629 [92], SRP111140, SRP130935 [93], SRP165232, SRP098826, SRP102403 [94], SRP154399, SRP090318 [95], DRP005173 [96], SRP107149, SRP245657, SRP149124, SRP060755, SRP163366 [97], SRP071243, SRP272124 [98], SRP103077, SRP163908, SRP170855 [99], SRP217227 [73], SRP132876 [100], SRP100408 [101], SRP105086, SRP076397, SRP182670, and SRP109036.

SRA projects processed for mouse are SRP217487 [102], SRP101928 [103], SRP075985 [104], SRP105082 [9], SRP165933 [41], SRP261290 [105], SRP118601 [106], SRP107774 [107, 108], SRP226118, SRP252213 [109], SRP229756, SRP250878, SRP131117 [110], SRP247488 [111], SRP223513 [112], SRP119332 [113], SRP268173 [67], SRP270993 [114], SRP179647 [115], SRP255620 [116], SRP100871 [117], SRP192917 [14], SRP156597 [118], SRP227097 [67], ERP114475, SRP249897 [119], SRP096571 [120], SRP144391 [121], SRP110616 [122], SRP292639 [119], SRP194410 [123], SRP200567 [124], and SRP218950 [118].

SRA projects processed for fly are ERP122732 [125], SRP165773 [126], SRP119928 [127], ERP112882, SRP050096 [128], SRP223221 [129], SRP097891 [130], ERP016479, SRP104256 [131], SRP186730 [132], SRP230396 [133], SRP107636 [134], SRP073988 [135], SRP111713 [136], SRP107637 [134], SRP195621 [84], SRP193880, SRP168946 [137], SRP107556 [134], SRP158369 [138], SRP110166, SRP165772 [139], ERP112723, SRP219433, SRP156199 [140], SRP199618 [141], SRP132075 [142], SRP140881, and SRP181908 [143].

In total, we aggregated 119 human projects, 33 mouse projects, and 29 fly projects. Additional file 2: Tables S1, S2 and S3 further summarize the number of runs, sequencing depth, and cell-type information for each project in respective species.

### Hi-C data processing pipeline

All runs were reprocessed from short read sequence data to reduce differential computational noise across experiments. Restriction enzymes were identified for each sample from the literature. SRA files were downloaded using prefetch, then converted to paired FASTQ files using fasterq-dump. FASTQ files were processed using the HiCUP tool [144], with the alteration that short reads were aligned using the STAR aligner, instead of the default Bowtie2 [145]. HiCUP truncates the reads based on restriction site, aligns them, and filters artifactual and duplicated data. Reads were aligned to the hg38, mm10, and dm6 in human, mouse, and fly respectively. Output SAM files were converted to indexed and compressed Pairs files using the bam2pairs tool. Finally, pairwise chromosome-chromosome contact matrices were generated at single base-pair resolution.

### Building chromatin contact matrix

To obtain a chromatin contact matrix, each chromosome is divided up into “bins” of a specific size. The number of base pairs in each bin represents the “resolution” of the matrix. The contact frequency for each bin pair is obtained by summing the reads falling in that bin. The chromatin contact matrix was generated at 8 resolutions (1KB, 5KB, 10KB, 25KB, 40KB, 100KB, 250KB, and 500KB) in cis for all species and trans for only fly. For human and mouse, trans chromatin contact matrices at 1-KB and 5-KB resolutions were not processed due to high memory requirements (more than 2TB). These files were written in HiCMatrix (https://github.com/deeptools/HiCMatrix) h5 format. For each species, we excluded sex chromosomes and considered only autosomes (human: chr1 to chr22, mouse:chr1 to chr19 and fly: chr2L, chr2R, chr3L, chr3R, chr4). The contact frequency of each genomic pair coordinate was summed across runs to generate a project-level chromatin contact matrix. The sequencing depth of a project in cis and trans is obtained by summing all the contacts in cis and trans respectively. The contact frequency was KR-normalized separately for the cis and trans networks to adjust for nonuniformities in coverage introduced due to experimental bias [146] using the hicCorrectMatrix tool of HiCexplorerV3.6 [147]. All project-level chromatin contact matrices within each species were further summated to create species-level meta-Hi-C maps. Gene transcription start site (TSS) and transcription end site (TES) were used to determine the bins in which the gene resides. A list of genes, TSS, and TES were obtained as GTF files from ENSEMBL (September 2019). To determine the contact frequency between each gene pair, we use the maximum contact frequency between each bin in which genes reside. This method was previously used by Babaei et al. [21].

### Coexpression data

The coexpression network used in this study is a “high confidence gene” aggregated coexpression network available at https://labshare.cshl.edu/shares/gillislab/resource/CoCoCoNet/ which was generated using the method previously described in CoCoCoNet [22]. In brief, several bulk RNA-seq datasets were obtained from NCBI’s SRA database (unique SRA Study IDs). Networks for each dataset are built by calculating the Spearman correlation between all pairs of genes, then ranking the correlation coefficients for all gene-gene pairs, with NAs assigned the median rank. Each network is then rank standardized and normalized by dividing by the maximum rank. Aggregate networks are then generated by averaging rank standardized networks from individual datasets. There are 23,465, 20,672, and 9636 genes (sex chromosomes are excluded) in the coexpression network for human, mouse, and fly.

### Compartment, subcompartment, and TAD assignment

Gene compartments were either identified using “hicPCA” (tool of HiCexplorerV3.6 [147] which is based on the Lieberman-Aiden et al. method [8] using each chromosome KR-normalized cis-contact matrix or Calder2.0 [23] available at github (https://github.com/CSOgroup/CALDER2.0). Gene density was used for A or B compartment assignment when the “hicPCA” tool was used. TADs and TAD boundaries were identified using TopDom [148]. A TAD is overlapping across two Hi-C experiments at a given resolution if the TAD’s start and end positions are the same in both experiments.

### eQTL data source and processing

A list of tissue-specific “significant” variant gene pair associations and “all” variant gene pair associations (including non-significant associations) across 54 tissues along with the distance between the variant and gene TSS (at bp resolution) were obtained from the GTEx Portal v8 at https://gtexportal.org. Since the meta-Hi-C network is not tissue-specific, we combined the data across tissues to generate a set of unique “significant” and “all” variant gene pair associations. To obtain a list of “non-significant” gene pair associations, “significant” variant gene pair associations were removed from “all” variant gene pair associations data. All variants in the coding regions and up to 1KB of any gene TSS and TES were removed. For performance score, 1-KB cis chromatin contact matrix is used, and for each eVariant, only genes in unique bins are tested. The total number of variants tested is 1,574,194 at 1-KB resolution. The total number of variant gene pair tested is 54,220,988 among which 4,319,205 pairs are significant.

### Cross-species analysis

A list of 1:1 orthologs for a pair of species was obtained from OrthoDB [149]. Species divergence time was sourced from Timetree [150].

## Supplementary Information

Additional file 1 Supplementary Figures S1-S5 with each legend. Fig S1. Meta-Hi-C network benchmarking in mouse. Fig S2. Meta-Hi-C network benchmarking in human. Fig S3. TAD coexpression vs contact coexpression. Fig S4. TAD and boundary conservation across experiments. Fig. S5. Contact coexpression conservation at various coexpression thresholds.

Additional file 2 Table S1. Details of each individual Hi-C project used for building human meta-Hi-C network. Table S2. Details of each individual Hi-C project used for building mouse meta-Hi-C network. Table S3. Details of each individual Hi-C project used for building fly meta-Hi-C network.

Additional file 3 Review history.

## Data Availability

The meta-Hi-C matrix for human, mouse, and fly are available for download from the online tool at https://gillisweb.cshl.edu/HiC/ or direct download at https://labshare.cshl.edu/shares/gillislab/resource/HiC/ or Figshare [151].
