# Supplementary material for: A global high-density chromatin interaction network reveals functional long-range and trans-chromosomal relationships
Source: Genome Biol. 2022 Nov 9;23:238. doi: 10.1186/s13059-022-02790-z (PMC9647974; doi:10.1186/s13059-022-02790-z)
Supplement: Supplementary file 1 — Additional file 1 Supplementary Figures S1-S5 with each legend. Fig S1. Meta-Hi-C network benchmarking in mouse. Fig S2. Meta-Hi-C network benchmarking in human. Fig S3. TAD coexpression vs contact coexpression. Fig S4. TAD and boundary conservation across experiments. Fig. S5. Contact coexpression conservation at various coexpression thresholds. [file 13059_2022_2790_MOESM1_ESM.pdf]

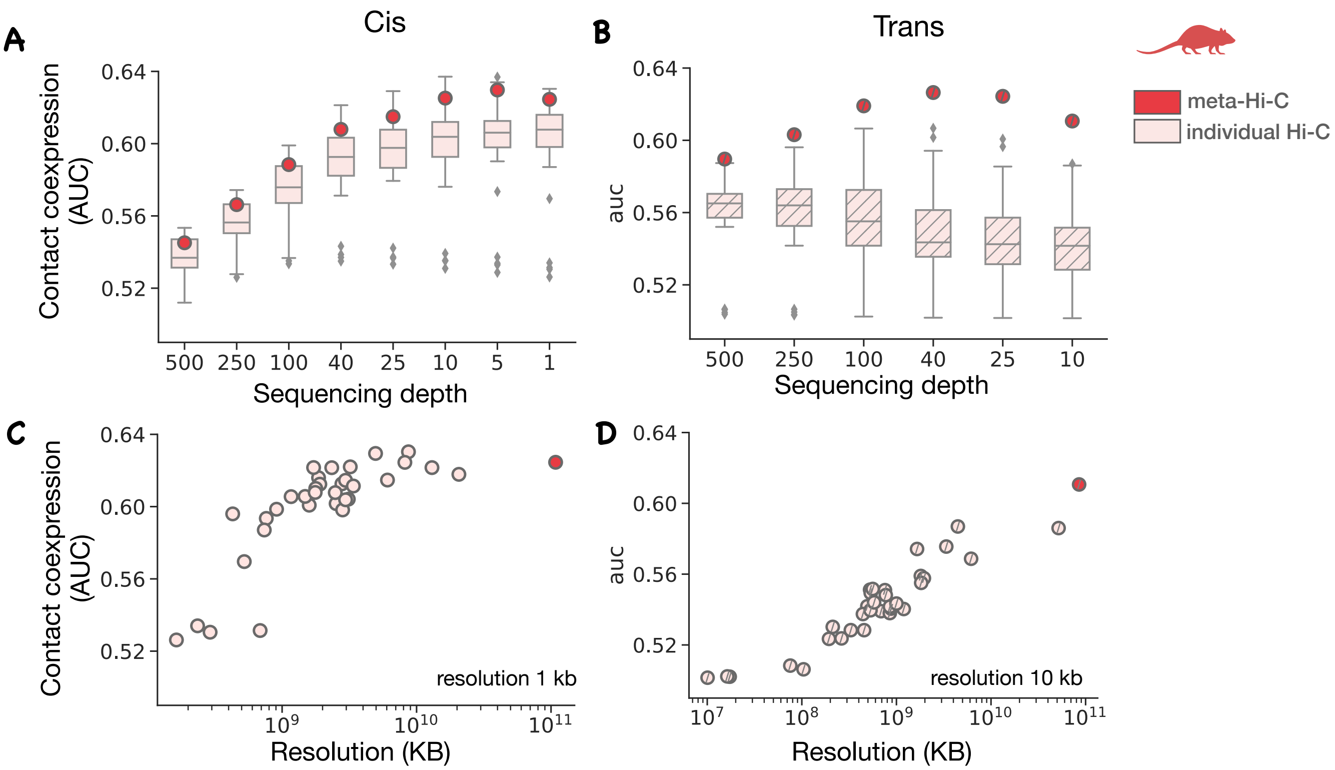

Fig S1. meta-Hi-C network benchmarking in Mouse. The boxplot shows the distribution of contact coexpression for each project at various resolutions in cis (A) and trans (B). Circles represent the performance of the cis meta-Hi-C network. Contact coexpression for individual and metaHi-C networks in cis (C) and trans (D) as a function of sequencing depth at 1KB resolution.

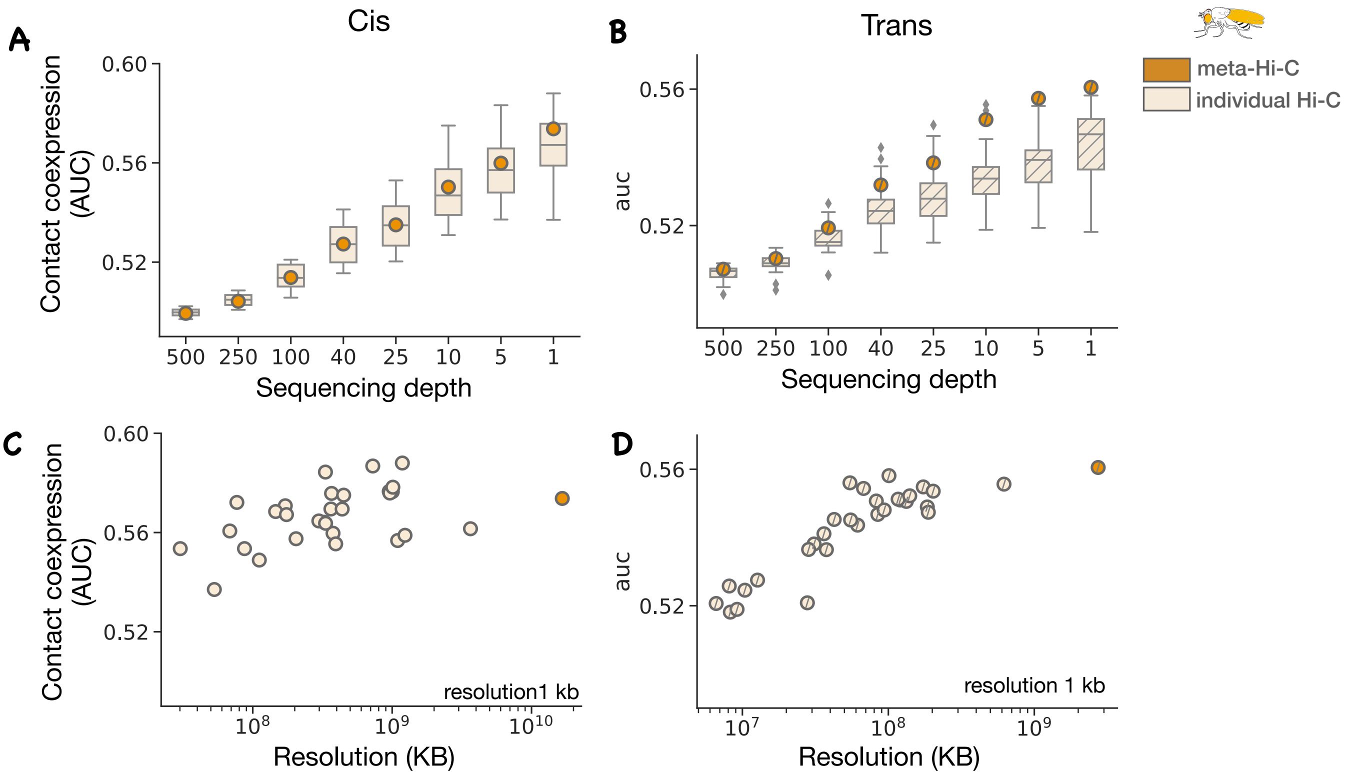

Fig S2. meta-Hi-C network benchmarking in Fly. The boxplot shows the distribution of contact coexpression for each project at various resolutions in cis (A) and trans (B). Circles represent the performance of the cis meta-Hi-C network. Contact coexpression for individual and metaHi-C network in cis (C) and trans (D) as a function of sequencing depth.

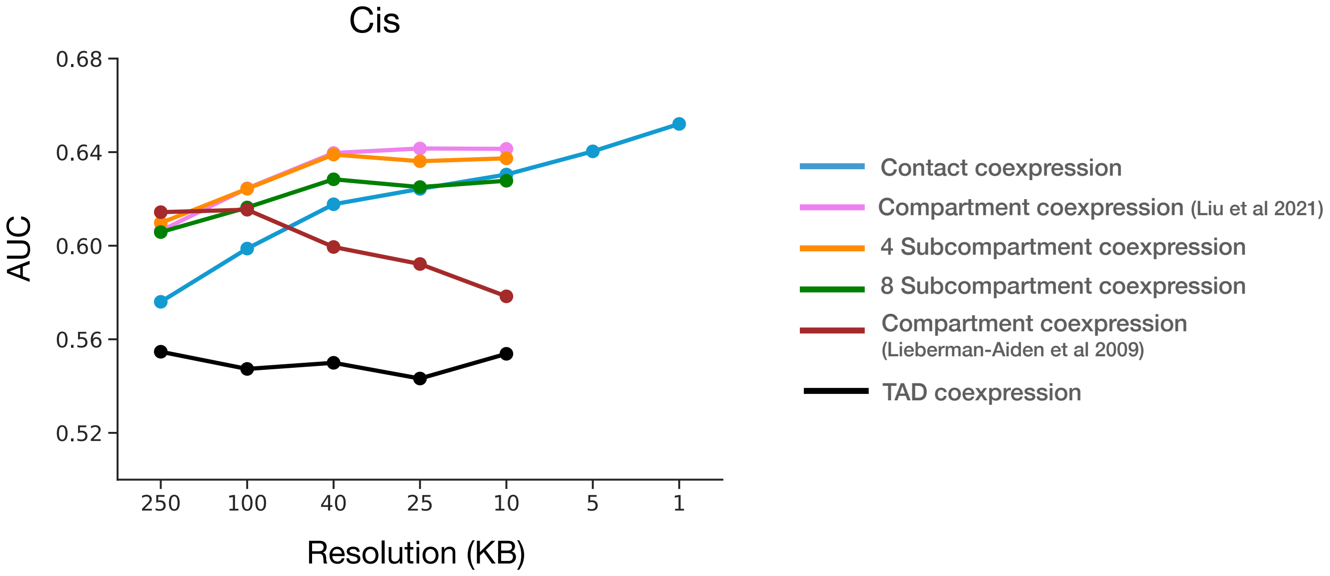

Fig S3. TAD coexpression vs contact coexpression. Comparison of contact coexpression score of meta-Hi-C network and compartment coexpression score at various resolutions. We called compartments in each individual network and then aggregated those calls, capturing the probability of sharing a compartment across data. The compartment coexpression metric captures the ability of aggregated compartment preference to predict coexpression. Subcompartment coexpression and TAD coexpression is defined analogously to compartment coexpression.

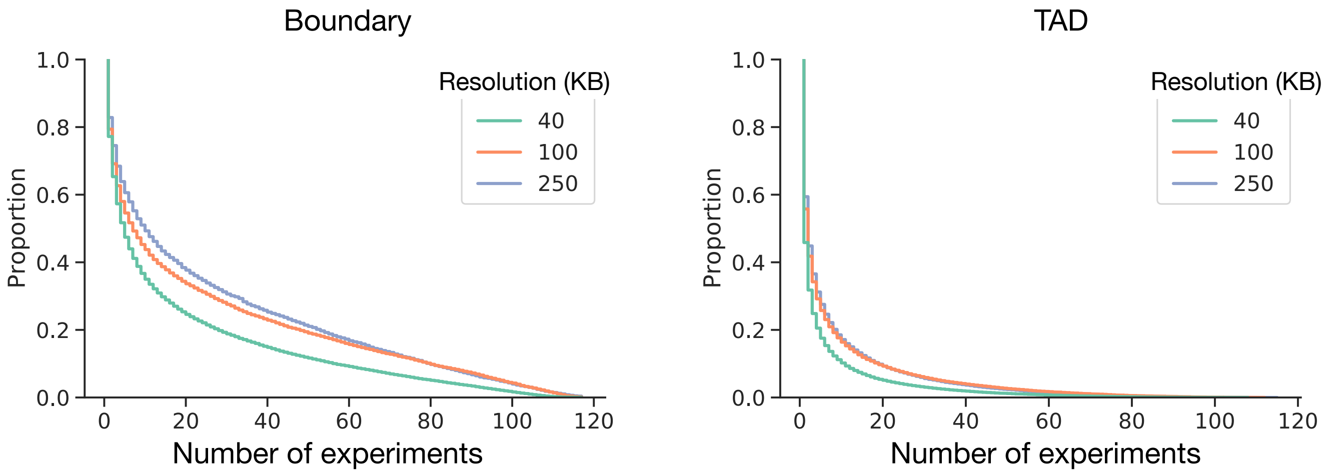

Fig S4. TAD and boundary conservation across experiments. The proportion of boundaries and TADs conserved across a given number of experiments or more from Hi-C networks at 40KB, 100KB, and 250KB resolution. The number of TADs conserved across experiments decreases relatively rapidly. While we did not find any TAD which was conserved across all the experiments, there is a small number present in a large proportion of the data.

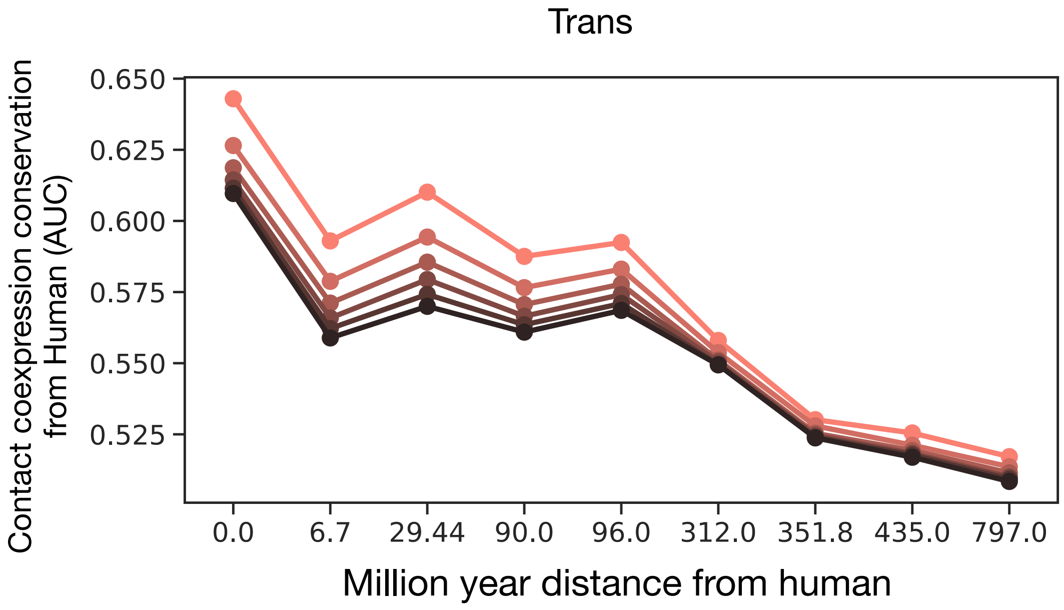

Fig S5. Contact coexpression conservation at various coexpression thresholds. We measured the “contact coexpression conservation” by ranking all the edges in the chromatin contact network of Human and then used it to predict the gene’s top 1, 5, 10, 15, 20 and 25 percent coexpressed gene pairs in other species. The plot below shows the median “contact coexpression conservation” AUC at various million year distance from Human. The color intensity decreases with increase in the fraction of gene’s top percent coexpressed gene pairs.
