# Supplementary material for: A global high-density chromatin interaction network reveals functional long-range and trans-chromosomal relationships
Source: Genome Biol. 2022 Nov 9;23:238. doi: 10.1186/s13059-022-02790-z (PMC9647974; doi:10.1186/s13059-022-02790-z)
Supplement: Supplementary file 3 — Additional file 3 Review history. [file 13059_2022_2790_MOESM3_ESM.docx]

Review History

**First round of review**

**Reviewer 1**

**Were you able to assess all statistics in the manuscript, including the appropriateness of statistical tests used?**

Yes, and I have assessed the statistics in my report.

**Comments to author:**

This manuscript uses a meta-analysis of many published Hi-C studies in three species to show that aggregation of Hi-C data across projects can yield insights into 3D chromatin architecture and its relation to gene expression. In particular, the empirical analysis here suggests that this meta-analysis yields improved power to predict co-expression, linkages to cis-regulatory elements, and cross-species relationships. Overall, this is a straightforward and compelling study, though of course I do have some suggestions for improvements.

Please change the terminology to use "Hi-C" instead of "3C" to refer to Hi-C experiments. The fields suffers a bit from an ambiguity in that "3C" can either mean a 3C experiment, as originally defined in Job Dekker's seminal paper, or can refer to the class of chromosome conformation capture methods (which includes 3C, 4C, 5C, etc.). As written, I thought you were aggregating 3C experiments (which seemed weird), and only realized my mistake on p. 3 line 41 (Incidentally, I am using your pagination and line numbers, rather than the ones added by the editorial system). Please change the terminology. I think this should be a "meta-Hi-C" matrix rather than "meta-3C," and earlier references (p. 1, line 8; p. 3, line 27) should be corrected.

In the second paragraph on p. 2, I was left wondering about resolution (aka "bin size"). It seems strange to have this entire discussion without mentioning that important axis.

You have omitted some interesting (and basic) statistics about the meta-3C map such as how much denser it is than the project-level maps, especially in the trans-contacts regions. Adding a visualization of (a portion of) the meta-map and comparing it to individual maps would be nice.

I couldn't figure out Figure 1D. The description doesn't make clear what this violin plot is. Are the elements in the plot projects? Is the horizontal axis a density? If so, why does it go both positive and negative? I think you should rotate the plot 90 degrees and not mirror the cis and trans. And of course improve the explanation of what we're looking at here.

The logical transition to the new paragraph at the top of page 6 is unclear. We are told that the motivation her is to "validate that the meta-3C network has more uniform coverage," but this left me wondering (1) what does it mean to have "uniform coverage," (2) more uniform than what, and most importantly (3) why is this an important question. It seems like a sentence or two of explanation/motivation is needed. The subsequent sentences sort of make the logic clear, but it took me a while to catch up.

It's worth noting that the notion of "resolution" is not even mentioned until p. 6, line 77. It should be mentioned and explicitly defined much earlier.

I don't think the compartment analysis beginning on page 6, line 89, makes sense. Compartments vary between cell types, so it doesn't really make sense to talk about compartments for this kind of meta-map. Furthermore, recent analysis suggests that compartments are sensitive to sequencing depth and that in practice nearly every active gene's promoter is in the A compartment (https://www.biorxiv.org/content/10.1101/2021.10.03.462599v2). I think this analysis should be eliminated.

To me, it seems striking that the human-fly and mouse-fly AUCs for the cross-species gene contact task are exactly chance (0.5, p. 9, line 148-149). This should be called out and at least briefly discussed. Presumably, this reflects the radically different 3D genome organization of the drosophila genome.

I did not find Figure 4 to be useful. This should be eliminated.

Minor:

Page 1, line 11: Rephrase the sentence beginning "Unlike individual experiments" to make clear that you are reporting, in this sentence, the results of an empirical comparison done here.

Page 1, line 20: This is the first mention of eQTLs. I think you should use consistent terminology in the preceding paragraph and in this sentence.

Page 3, line 27: Write out "conformation capture (CC)" the first time you use this abbreviation.

Page 4, line 43-44: Do we need to know about the abbrevations "SRR" and "SRP"? They don't seem to be used again. If you need them, then write them out the first time they are used.

Throughout: Species names (e.g., "human") are not capitalized.

Page 4, line 48-49: I was curious how the mapping to genes was done. I think the main text should mention that this involves a fixed-bin matrix followed by taking a max over all bins within a given gene.

Page 4, line 51: chromosome -> chromosomes

Page 4, line 54: colocalized [genes] to be

Page 6, lines 80-88: The figure references are messed up here.

Page 6, line 84: the in trans -> the trans.

**Reviewer 2**

**Were you able to assess all statistics in the manuscript, including the appropriateness of statistical tests used?**

No, I do not feel adequately qualified to assess the statistics.

**Comments to author:**

Chromosome conformation capture (3C)-based technologies such as Hi-C are often limited in resolution due to high sequencing depth required to capture all pairwise contacts between chromosome fragments in a given species. Here, the authors propose a meta-analysis approach, dubbed meta-3C, by aggregating an impressive number of available of Hi-C data, i.e. 3600 Human (119 projects Human projects, 6700 Mouse (33 Mouse projects), and 500 Fly 3C experiments (29 Fly projects), leading to 304 billion, 193 billion, and 19 billion contacts in the respective species, which largely outperform Hi-C experiments when taken individually. The idea here is do generate a ressource to have a much better resolution for the identification of cis- and trans- contacts, but on the other side, with the lack of cell-type specific interactions. However, the authors showed by addressing different parameters that meta-3C networks - in both cis and trans contacts - perform better (sometimes much better) than individual 3C networks. Some of the measured parameters with meta-3C bring new insights in the comprehension of long-range and trans chromosomal interactions and could be applied to a wide range of genomics research.

I found this paper well organized and presented, and useful for the genomic community. Importantly, the authors have made their meta-3C Hi-C aggregates available for the community via an online tool, at least the raw data are easily accessible and can be converted using HiCExplorer. I have however some comments that could be addressed to improve the manuscript.

Major points

- The authors measured the ability of interaction frequency to predict the gene's top 1% coexpression partners, named « contact coexpression ». In the meta-3C network, an AUC score of 0.65 is obtained for both cis- and trans contacts (Fig. 2B-C). What does it mean exactly in term of quantification? Does it mean that cis and trans interactions are equivalent in term of « contact coexpression ». Is there a specific normalization between cis and trans contacts?

- To look at large genomic scale, they defined « compartment coexpression » and they found that compartment co-expression performance is a small fraction of contact co-expression, suggesting more complex sub-compartmental relationships or that other trans interactions contribute. Here, the genome sub-division in compartment A and B may represent a too low resolution for defining contact coexpression.

It would have been nice to look at lower scale than compartments, such as Topologically-Associating domains (TAD).

- Actually, since they have aggregating many different Hi-C projects from various cell origin, do they still have a good definition of TAD and boundaries? TADs and barriers are supposed to be relatively stable between cell types, at least at relatively low resolution. Could they define invariant TAD region among, i.e. stable in all the datasets ?

- Could the author define a « TAD coexpression » score. Do TADs perform better than compartment for contact co-expression?

This aspect is important since TADs are often considered as functional genomic units inside which genes could be co-expressed. Is this a rule or more an exception?

- On the same trend, the authors could have also consider epigenetic sub-compartments as defined by Rao et al (doi: 10.1016/j.cell.2014.11.021) or the multi-scale chromatin sub-compartments as recently defined by Liu et al using the Calder algorithm (https://doi.org/10.1038/s41467-021-22666-3). This could also have been used to define « sub-compartment coexpression » in comparaison with « compartment coexpression ».

Looking at a smaller genomic scale, i.e. TADs or sub-compartments, may be more appropriate to define co-expression.

- Concerning the type of cells used to generate Hi-C maps, it would be nice to have an idea of the type of cells that was used in each project, whether they are pluripotent cells, differentiated cells, immortalized cells, cells from tissues… I think this could be integrated in their Supplementary tables of projects.

- Concerning cell-type, did the authors have tried aggregating Hi-C data from the same cell type? This approach could also performs well at multi-scale compare to individual network and could definitely be useful for the community.

- It is somewhat surprising that they didn't use the Hi-C data generated by Bonev et al (doi: 10.1016/j.cell.2017.09.043), which had a very profound sequencing depth both in ESC and neuronal cells. For the mouse meta-3C, this could have been a better dataset than others with less profound sequencing depth.

- As mentioned by the authors, the distance between the variant and gene transcription start site (TSS) remains almost the only metric widely used to annotate target genes for variants (define here as « Proximity CRE »). This approach is highly limited and meta-3C networks could be a very attractive alternative method to determine the target gene of specific genetic elements (define here as « Contact-CRE »).

When measuring the AUC score of contact-CRE, they find that meta-3C network outperforms individual networks at all distance thresholds, but more importantly, the « contact CRE » meta-3C performed much better than the « proximity CRE » at large distance (> 400000).

This is an important result, but I found this part a bit confusing. Actually why « proximity CRE » is attributed to « meta-3C » in Fig.2i, since it is the linear genomic distance that define this parameter, and not HiC contacts (if I understood well).

In other words, this paragraph would gained in clarifying the notion of « contact CRE » versus « proximity CRE » to reinforce the potential of meta-3C in predicting variant gene pair associations.

- Actually, it could also have been nice to have few representative examples of contact-CRE versus proximity CRE to illustrate the potential of meta-3C network in predicting variant gene pair associations, more efficiently than by linear genomic distance.

- When measuring "contact coexpression conservation", by ranking all edges in the chromatin contact network of one species and then used to predict the gene's top 10% of coexpressed gene pairs in another species. What would happen in taking the Top 1%, 5%, 20%…?

In other words, are the strongest contact coexpression the most conserved?

- Concerning evolutionary conservation, some Hi-C data (individual 3C network) are available for some species used in this study. Could they test their prediction of "contact coexpression conservation"?

This could be a real asset of their meta-3C.

Minor points

- In Figure 3D, when comparing meta-3C and individual projects, not sure if we are looking at the cis or trans networks. This should be precised.

- The authors should indicate how many variant gene pair associations were used in their study. The information is not easy to find.

- Same for gene's top 1%, top 10% of co-expression partners… How many genes in these cases?

- For defining eQTL interactions, the term « CRE » which stands for « cis-regulatory elements » is a bit confusing here, because he doesn't contain the notion of genetic variants. For the sake of clarity, I would change this acronym.

- There is a mistake in Table S3: Details of each individual project used for building Mouse meta-3C network -> for building the Drosophila meta-3C network.

**Authors Response**

**Point-by-point responses to the reviewers’ comments:**

Reviewer #1:

This manuscript uses a meta-analysis of many published Hi-C studies in three species to show that aggregation of Hi-C data across projects can yield insights into 3D chromatin architecture and its relation to gene expression. In particular, the empirical analysis here suggests that this meta-analysis yields improved power to predict co-expression, linkages to cis-regulatory elements, and cross-species relationships. Overall, this is a straightforward and compelling study, though of course I do have some suggestions for improvements.

- Please change the terminology to use "Hi-C" instead of "3C" to refer to Hi-C experiments. The fields suffers a bit from an ambiguity in that "3C" can either mean a 3C experiment, as originally defined in Job Dekker's seminal paper, or can refer to the class of chromosome conformation capture methods (which includes 3C, 4C, 5C, etc.). As written, I thought you were aggregating 3C experiments (which seemed weird), and only realized my mistake on p. 3 line 41 (Incidentally, I am using your pagination and line numbers, rather than the ones added by the editorial system). Please change the terminology. I think this should be a "meta-Hi-C" matrix rather than "meta-3C," and earlier references (p. 1, line 8; p. 3, line 27) should be corrected.

*Reply: We apologize for the confusion. We agree that “meta-Hi-C” is more appropriate term and we have replaced meta-3C everywhere in the manuscript with meta-Hi-C.*

- In the second paragraph on p. 2, I was left wondering about resolution (aka "bin size"). It seems strange to have this entire discussion without mentioning that important axis.

*Reply: We appreciate the pointer and to improve clarity we have made the following changes:*

*● Have added the following in introduction to introduce resolution*

*From Hi-C experiments a nxn chromatin contact matrix is generated where the genome is divided into n equally sized bins and contact frequency between each bin-pair is obtained by summing the pair-ended reads spanning between a pair of bins (Fig 1 a). The bin size is also referred to as 'resolution' and is dependent on the sequencing depth of the experiment—at low sequencing depth the matrix is generated using large bin sizes (low resolution) to reduce the sparsity of the matrix. The commonly used sizes of these bins can range from 1 kb to 1 Mb.*

*● New Panel was added to Figure 1 where we define resolution*

*Fig 1: Creating meta-Hi-C network. A) Genes are co-localized in chromatin 3D structure through frequent chromatin contacts. Each chromosome is divided up into “bins” of a specific size referred to as resolution and the chromatin contact matrix represents the number of pair-ended reads spanning between a pair of bins. The contact matrix can be represented with networks where nodes are genes and edges are interaction frequencies. The cis and trans networks consist of intra-chromosome and inter-chromosome edges respectively.*

- You have omitted some interesting (and basic) statistics about the meta-3C map such as how much denser it is than the project-level maps, especially in the trans-contacts regions. Adding a visualization of (a portion of) the meta-map and comparing it to individual maps would be nice.

*Reply: We’ve made additions to address this point. Some of them are quite compelling, so we are grateful it was raised.*

*● We have added Fold enrichment metric in Figure 1F and Figure 1G to compare the enrichment of contacts in the meta-Hi-C network for a gene when compared with maximum contact or median contact of that gene in any individual HiC experiment. This highlights the comprehensively greater depth of the meta-HiC network.*

*Fig1: D) Total number of contacts in cis and trans meta-Hi-C network of Human, Mouse, and Fly. n, N are the number of runs and projects aggregated in each species respectively. E) Contact density (total contacts/number of 1bp bins) across individual projects in cis and trans for each species. Individual points are individual experiments and the darker shades of points are the values for the meta-Hi-C network. Distirbution of fold enrichment of contacts among genes in meta-Hi-C network relative to maximum (F) or median (G) number of contacts for that gene among individual network in cis and trans. Total number of genes in F and G are 23,465, 20,672 and 9,636 in Human, Mouse and Fly respectively.*

*● We have added a visualization comparing individual and meta-Hi-C network as shown in Figure 1C, below.*

*Fig 1: C) Visual comparison of our meta-Hi-C and two individual Hi-C contact matrices: Vian et al 2018 \cite{Vian2019} (a typical experiment) and Rao et al 2014 \cite{Rao2014} (densest experiment in trans) at 100KB (top) and 25KB resolution (bottom). The maximum contact frequency is given at the bottom of each map. The high intensity region observed in our meta-Hi-C contact matrix at 25KB resolution belongs to gene UBEJ2 on Chr1 and ZFP64 on Chr20. Both these genes are also strongly co-expressed in our co-expression network with a co-expression strength of 0.98 on a genome-wide rank-standardized scale of 0 to 1.*

- I couldn't figure out Figure 1D. The description doesn't make clear what this violin plot is. Are the elements in the plot projects? Is the horizontal axis a density? If so, why does it go both positive and negative? I think you should rotate the plot 90 degrees and not mirror the cis and trans. And of course improve the explanation of what we're looking at here.

*Reply: We apologize for the confusing plot here. We have updated figure 1E (was previously fig 1D) as follows:*

*Instead of violin plots we have added scatter plot with contact density in cis and trans for each species (Figure 1E). Individual points are individual experiments and the darker shades of points are the values for the meta-Hi-C network in their respective species, highlighting the higher performance.*

*Fig 1: E) Contact density (total contacts/number of 1bp bins) across individual projects in cis and trans for each species. Individual points are individual experiments and the darker shades of points are the values for the meta-Hi-C network. Distirbution of fold enrichment of contacts among genes in meta-Hi-C network relative to maximum (F) or median (G) number of contacts for that gene among individual network in cis and trans. Total number of genes in F and G are 23,465, 20,672 and 9,636 in Human, Mouse and Fly respectively.*

- The logical transition to the new paragraph at the top of page 6 is unclear. We are told that the motivation her is to "validate that the meta-3C network has more uniform coverage," but this left me wondering (1) what does it mean to have "uniform coverage," (2) more uniform than what, and most importantly (3) why is this an important question. It seems like a sentence or two of explanation/motivation is needed. The subsequent sentences sort of make the logic clear, but it took me a while to catch up.

*Reply: This is a helpful point. We realized that the term “uniform coverage” is misleading and hence we have removed it from the text; we meant something more like “more comprehensive”. We have added the following sentences:*

*Hi-C matrix in cis have high density of contacts at bins near the diagonal and the contact density decreases exponentially as the distance between the bins increases therefore Hi-C networks with high contact density can still have high sparsity at distant bins. This makes it difficult to capture functional contacts between distant gene pairs. Although cis meta-Hi-C network does not perform better than all individual network, unlike in trans, we still hypothesized that cis meta-Hi-C network have addional coverage for distant gene pairs. To test our hypothesis we compared the contact coexpression of individual Hi-C networks and meta-Hi-C networks at various linear distance thresholds in cis.*

- It's worth noting that the notion of "resolution" is not even mentioned until p. 6, line 77. It should be mentioned and explicitly defined much earlier.

*Reply: We have introduced resolution in the introduction*

*From Hi-C experiments a nxn chromatin contact matrix is generated where the genome is divided into n equally sized bins and contact frequency between each bin-pair is obtained by summing the pair-ended reads spanning between a pair of bins. The bin size is also referred to as 'resolution' and is dependent on the sequencing depth of the experiment—at low sequencing depth the matrix is generated using large bin sizes (low resolution) to reduce the sparsity of the matrix. The commonly used sizes of these bins can range from 1 kb to 1 Mb.*

*We have also added the following sentences in the results section:*

*The resolution of Hi-C matrix is an important parameter for obtaining gene networks and hence we evaluated the contact coexpression of individual Hi-C and meta-Hi-C networks at several resolutions.*

- I don't think the compartment analysis beginning on page 6, line 89, makes sense. Compartments vary between cell types, so it doesn't really make sense to talk about compartments for this kind of meta-map. Furthermore, recent analysis suggests that compartments are sensitive to sequencing depth and that in practice nearly every active gene's promoter is in the A compartment (https://www.biorxiv.org/content/10.1101/2021.10.03.462599v2). I think this analysis should be eliminated.

*Reply: We apologize for the confusion here. We agree that calling compartments in the meta network would not make (much) sense, and that is not what we did. Instead, we called compartments in each individual network and then aggregated those calls, capturing the probability of sharing a compartment across all data. We wanted to assess whether this would account for the trans relationships we saw. We have clarified the text.*

*In each individual network, we identified the compartment of each gene and then binarized the compartment preference for each gene pair: so each experiment becomes a binary network of gene pair found in the same compartment (1) or a different compartment (0). The binarized individual networks are then aggregated, capturing the probability of sharing a compartment across all data. For each gene, we measure the ability of aggregated compartment frequency to predict the gene’s top 1% coexpression partners. We call this measure “compartment coexpression” and is expressed as an AUC with possible values ranging between 0 and 1.*

- To me, it seems striking that the human-fly and mouse-fly AUCs for the cross-species gene contact task are exactly chance (0.5, p. 9, line 148-149). This should be called out and at least briefly discussed. Presumably, this reflects the radically different 3D genome organization of the drosophila genome.

*Reply: We agree and we have added the following in the discussion.*

*Additionally, we find that trans chromosomal contacts show evidence of conservation between Human and Mouse. In contrast, We do not find any conservation between these mammalian networks and Fly (Human-Fly AUC 0.50, Mouse-Fly AUC 0.51). This is striking in its nearly exact correspondence with the null, but the very large amount of data combined with the dramatically different genomes appears to leave no average signal when analyzed across homologues. It remains plausible that a model trained specifically to account for the broad cross-species differences would find subtler biological overlaps our direct assessments miss.*

- I did not find Figure 4 to be useful. This should be eliminated.

*Reply: We have removed this figure.*

Minor:

Page 1, line 11: Rephrase the sentence beginning "Unlike individual experiments" to make clear that you are reporting, in this sentence, the results of an empirical comparison done here.

*Reply: Rephrased sentence:*

*We find that while individual dataset networks are essentially unable to predict any long range co-expression (median 0.54 AUC), meta-HiC perform comparably in both cis and trans (0.65 AUC vs 0.64 AUC).*

Page 1, line 20: This is the first mention of eQTLs. I think you should use consistent terminology in the preceding paragraph and in this sentence.

*Reply: For consistency we have replaced cis-regulatory interactions by eQTLs in the preceding paragraph and everywhere in the text.*

Page 3, line 27: Write out "conformation capture (CC)" the first time you use this abbreviation.

*Reply: Thank you for pointing it out. We have replaced CC map in the manuscript with chromatin contact matrix.*

Page 4, line 43-44: Do we need to know about the abbrevations "SRR" and "SRP"? They don't seem to be used again. If you need them, then write them out the first time they are used.

*Reply: Thank you for pointing it out. We have removed SRR and SRP abbreviations since they are not needed.*

Throughout: Species names (e.g., "human") are not capitalized.

*Reply: Thank you for pointing it out. Have done so in text and figures.*

Page 4, line 48-49: I was curious how the mapping to genes was done. I think the main text should mention that this involves a fixed-bin matrix followed by taking a max over all bins within a given gene.

*Reply: Have added the following: To determine contact frequency between each gene pair we use the maximum contact frequency between each bin in which genes reside (see Methods).*

Page 4, line 51: chromosome -> chromosomes.

*Reply: Have replaced it.*

Page 4, line 54: colocalized [genes] to be

*Reply: As our first performance test, we assessed the tendency for spatially co-localized genes to be*

*coexpressed.*

Page 6, lines 80-88: The figure references are messed up here.

*Reply: Have addressed this.*

Page 6, line 84: the in trans -> the trans.

*Reply: Have addressed this.*

Reviewer #2:

Chromosome conformation capture (3C)-based technologies such as Hi-C are often limited in resolution due to high sequencing depth required to capture all pairwise contacts between chromosome fragments in a given species. Here, the authors propose a meta-analysis approach, dubbed meta-3C, by aggregating an impressive number of available of Hi-C data, i.e. 3600 Human (119 projects Human projects, 6700 Mouse (33 Mouse projects), and 500 Fly 3C experiments (29 Fly projects), leading to 304 billion, 193 billion, and 19 billion contacts in the respective species, which largely outperform Hi-C experiments when taken individually. The idea here is do generate a ressource to have a much better resolution for the identification of cis- and trans- contacts, but on the other side, with the lack of cell-type specific interactions. However, the authors showed by addressing different parameters that meta-3C networks - in both cis and trans contacts - perform better (sometimes much better) than individual 3C networks. Some of the measured parameters with meta-3C bring new insights in the comprehension of long-range and trans chromosomal interactions and could be applied to a wide range of genomics research.

I found this paper well organized and presented, and useful for the genomic community. Importantly, the authors have made their meta-3C Hi-C aggregates available for the community via an online tool, at least the raw data are easily accessible and can be converted using HiCExplorer. I have however some comments that could be addressed to improve the manuscript.

Major points

- The authors measured the ability of interaction frequency to predict the gene's top 1% coexpression partners, named « contact coexpression ». In the meta-3C network, an AUC score of 0.65 is obtained for both cis- and trans contacts (Fig. 2B-C). What does it mean exactly in term of quantification? Does it mean that cis and trans interactions are equivalent in term of « contact coexpression ». Is there a specific normalization between cis and trans contacts?

*Reply: We think this is an excellent question! In our analyses, a direct normalization is avoided by separating all analyses into purely cis and purely trans. This was done principally to avoid having a specific weighting to account for the difference (since the actual range of values is quite different) but we think it ultimately highlights a very compelling categorical distinction in our results. We agree that the similarity in performance implies that if standardizing to optimize HiC for co-expression prediction, there would be a possible weighting (perhaps 2:1 - i.e., downweighting cis). However, that’s taking co-expression as the gold standard. While we think it is a useful bronze standard to evaluate performances genome-wide, we prefer not to specifically optimize to fit to it. There is also the complication that AUROCs are an effect size and the space being assessed is different in cis and trans. We have lightly altered the text to call out the observation more.*

*Although contact coexpression scores in cis and trans are similar (0.63 AUC cis vs 0.64 AUC trans at 10KB resolution) the search space in trans is at least three times larger when compared to cis, making direct comparison of the aggregate “strength” of cis and trans relationships non-trivial.*

- To look at large genomic scale, they defined « compartment coexpression » and they found that compartment co-expression performance is a small fraction of contact co-expression, suggesting more complex sub-compartmental relationships or that other trans interactions contribute. Here, the genome sub-division in compartment A and B may represent a too low resolution for defining contact coexpression.

It would have been nice to look at lower scale than compartments, such as Topologically-Associating domains (TAD).

*Reply: We thank the reviewer for suggesting these tests which motivated some new experiments and interesting results, described in the more detailed response below.*

- Actually, since they have aggregating many different Hi-C projects from various cell origin, do they still have a good definition of TAD and boundaries? TADs and barriers are supposed to be relatively stable between cell types, at least at relatively low resolution. Could they define invariant TAD region among, i.e. stable in all the datasets?

*Reply: As suggested we identified boundaries and TADs using TopDom (Shin et al 2016) for every individual Hi-C network in Human. Below we show that proportion of boundaries and TADs conserved across a given number of experiments or more from Hi-C networks at 40KB, 100KB and 250KB resolution. The number of TADs conserved across experiments decreases relatively rapidly. While we did not find any TAD which conserved across all the experiments, there are a small number present in a large proportion of the data.*

*Method: TADs are conserved between two Hi-C experiments if both the boundaries of a TAD are simultaneously observed in both experiments. TADs are called using TopDom at various resolutions. A TAD is overlapping across two Hi-C experiments at a given resolution if the TAD’s start and end position are the same in both the experiments.*

- Could the author define a « TAD coexpression » score. Do TADs perform better than compartment for contact co-expression?

This aspect is important since TADs are often considered as functional genomic units inside which genes could be co-expressed. Is this a rule or more an exception?

*Reply: Yes, we defined TAD coexpression score analogously to compartment coexpression score: In each individual network, we identified the TAD of each gene and then binarized the TAD preference for each gene pair: so each experiment becomes a binary network of gene pair found in the same TADs (1) or a different TAD (0). For each gene, we measure the ability of aggregated TAD frequency to predict the gene’s top 1\% coexpression partners (Figure \ref{fig:s3}). We call this measure “TAD coexpression” and is expressed as an AUC with possible values ranging between 0 and 1.:*

*Below we compare TAD coexpression, compartment coexpression and contact coexpression at various resolution of Hi-C network in cis. We find that TAD coexpression score is lower than contact or compartment since TADs do not capture long-range interactions (Average TAD size is smaller than 1MB).*

- On the same trend, the authors could have also consider epigenetic sub-compartments as defined by Rao et al (doi: 10.1016/j.cell.2014.11.021) or the multi-scale chromatin sub-compartments as recently defined by Liu et al using the Calder algorithm (https://doi.org/10.1038/s41467-021-22666-3). This could also have been used to define « sub-compartment coexpression » in comparaison with « compartment coexpression ».

Looking at a smaller genomic scale, i.e. TADs or sub-compartments, may be more appropriate to define co-expression.

*Reply: We think that this is an excellent point. We previously used Lib et al method to define 2 compartments. We used Calder for defining 2 compartments and further 4 and 8 subcompartments. Below, we compare the compartment coexpression from the two methods (Calder(Liu et al 2021) and Lieberman-Aiden et al 2009) , 4-subcompartment-coexpression, 8-subcompartment-coexpression and contact-coexpression using trans Hi-C networks at several resolutions. We find that subcompartment coexpression is a small fraction of contact coexpression suggesting other trans interactions contribute (or compartment calling methods inadequately capture statistical relationships). We have added the results in Figure 2G.*

*Fig 2: G) Comparison of contact coexpression score of meta-Hi-C network and compartment coexpression score at various resolutions. We called compartments in each individual network and then aggregated those calls, capturing the probability of sharing a compartment across data. The compartment coexpression metric captures the ability of aggregated compartment preference to predict coexpression. Subcompartment coexpression is defined analogously to compartment coexpression. Compartments were called either using Liu et al 2021 method or Liberman-Aiden et al 2009 method.*

- Concerning the type of cells used to generate Hi-C maps, it would be nice to have an idea of the type of cells that was used in each project, whether they are pluripotent cells, differentiated cells, immortalized cells, cells from tissues… I think this could be integrated in their Supplementary tables of projects.

*Reply: Thank you for requesting this information. We have added the cell-type information whenever available for each project in supplementary tables S1, S2 and S3.*

- Concerning cell-type, did the authors have tried aggregating Hi-C data from the same cell type? This approach could also performs well at multi-scale compare to individual network and could definitely be useful for the community.

*Reply: We did not directly try this but do leave it open as an interesting experiment for the future. We agree that cell-type differences would be an important category but evaluation would be non-trivial - because the data would be more specific but also less well powered. I.e., it yields noisier cell-type networks in which most differences could be driven by power (+ quality control of data, etc). We are keen to engage in this problem in the future but think it is outside of the scope of the current contribution.*

- It is somewhat surprising that they didn't use the Hi-C data generated by Bonev et al (doi: 10.1016/j.cell.2017.09.043), which had a very profound sequencing depth both in ESC and neuronal cells. For the mouse meta-3C, this could have been a better dataset than others with less profound sequencing depth.

*Reply: We appreciate this pointer. Currently, we have frozen the meta-Hi-C network and have made it available via the online tool. We propose to have iterative updating of the meta-Hi-C network in future as more Hi-C networks become available (or we observe more existing data).*

- As mentioned by the authors, the distance between the variant and gene transcription start site (TSS) remains almost the only metric widely used to annotate target genes for variants (define here as « Proximity CRE »). This approach is highly limited and meta-3C networks could be a very attractive alternative method to determine the target gene of specific genetic elements (define here as « Contact-CRE »). When measuring the AUC score of contact-CRE, they find that meta-3C network outperforms individual networks at all distance thresholds, but more importantly, the « contact CRE » meta-3C performed much better than the « proximity CRE » at large distance (> 400000).

This is an important result, but I found this part a bit confusing. Actually why « proximity CRE » is attributed to « meta-3C » in Fig.2i, since it is the linear genomic distance that define this parameter, and not HiC contacts (if I understood well).

In other words, this paragraph would gained in clarifying the notion of « contact CRE » versus « proximity CRE » to reinforce the potential of meta-3C in predicting variant gene pair associations.

*Reply: Thanks for this correction. We have updated the label.*

- Actually, it could also have been nice to have few representative examples of contact-CRE versus proximity CRE to illustrate the potential of meta-3C network in predicting variant gene pair associations, more efficiently than by linear genomic distance.

*Reply: Excellent point. We have added the following example in Figure 3D:*

*Fig3: D) An example comparing contact-eQTL with proximity-eQTL. All genes associated with e-Variant rs142559680 along with their sequence position on X axis. The vertical separation between gene and variant increases as the distance between the variant and gene TSS increases. The thickness of the variant gene edge increases with increase in the contact frequency in the meta-Hi-C network. Although gene RP11-479A21.1 is the closest to the variant, gene RBM20 has strongest contact with the variant and also the only significantly associated gene with the variant.*

- When measuring "contact coexpression conservation", by ranking all edges in the chromatin contact network of one species and then used to predict the gene's top 10% of coexpressed gene pairs in another species. What would happen in taking the Top 1%, 5%, 20%…?

In other words, are the strongest contact coexpression the most conserved?

*Reply: Yes, as expected the stronger the co-expression, the higher the conservation. We measured the “contact coexpression conservation” by ranking all the edges in the chromatin contact network of human and then used to predict the gene’s top 1, 5, 15, 20 and 25 percent coexpressed gene pairs in the other species. The plot below shows the median “contact coexpression conservation” AUC at various million year distance from human. The color intensity decreases with increase in fraction of gene’s top percent coexpressed gene pairs.*

- Concerning evolutionary conservation, some Hi-C data (individual 3C network) are available for some species used in this study. Could they test their prediction of "contact coexpression conservation"?

This could be a real asset of their meta-3C.

*Reply: Yes, that is true that there are Hi-C data available for few other species but unfortunately the number of Hi-C data are relatively few. Our strategy really does rely on having quite a lot of data, especially since we want these to have general utility for other researchers. We do propose to update as new species become available. For example, uur co-expression network list has risen in a few years from a dozen or so reference species to approximately 40.*

Minor points

- In Figure 3D, when comparing meta-3C and individual projects, not sure if we are looking at the cis or trans networks. This should be precised.

*Reply: Thank you. We have added the appropriate labels (cis or trans) in all plots.*

- The authors should indicate how many variant gene pair associations were used in their study. The information is not easy to find.

*Reply: Total number of variants tested are 1,574,194 at 1KB resolution. Total number of variant gene pair tested are 54,220,988 among which 4,319,205 pairs are significant.*

- Same for gene's top 1%, top 10% of co-expression partners… How many genes in these cases?

*Reply: Total number of genes in the Human coexpression network are 23,465. Top 1% and top 10% of co-expressioin partners in genome-wide network excluding itself is 234 genes and 2346 genes respectively.*

*Total number of genes in the Mouse coexpression network are 20,672. Top 1% and top 10% of co-expressioin partners in genome-wide network excluding itself is 206 genes and 2067 genes respectively.*

*Total number of genes in the Fly coexpression network are 9,636.*

*Top 1% and top 10% of co-expressioin partners in genome-wide network excluding itself is 96 genes and 963 genes respectively. Top 1% or 10% of co-expression partners in cis and trans network vary depeding on the chromosome the gene lies in.*

- For defining eQTL interactions, the term « CRE » which stands for « cis-regulatory elements » is a bit confusing here, because he doesn't contain the notion of genetic variants. For the sake of clarity, I would change this acronym.

*Reply: We have removed the term CRE from the text and replaced it with eQTL.*

- There is a mistake in Table S3: Details of each individual project used for building Mouse meta-3C network -> for building the Drosophila meta-3C network.

*Reply: Thanks. We have updated it.*

**Second round of review**

**Reviewer 1**

Overall, you have done a good job of responding to my previous reviews.

I was, however, confused by this sentence in the revision: "Although cis meta-Hi-C network does not perform better than all individual network, unlike in trans, we still hypothesized that cis meta-Hi-C network have additional coverage for distant gene pairs." Specifically, I can't figure out what you mean by "perform better" here: perform better at what task? Also, the hypothesis is not clear to me: "cis meta-Hi-C network have additional coverage for distant gene pairs." Additional coverage relative to what? I think you mean that co-expressed genes have higher coverage in the meta-Hi-C network than non-co-expressed genes. The text needs to be clarified here.

The compartment analyis is clearer now. However, I think this sentence is misleading: "We find that compartment coexpression and subcompartment coexpression performance is a small fraction of contact coexpression, suggesting ..." It looks to me like the AUC decreases from around 0.65 to around 0.60. I would hardly characterize this as a "small fraction." The difference in performance does support the conclusion that other factors are at play, but the text should more accurately reflect the observed results.

**Reviewer 2**

The authors adequately address my critical points.

I am definitely favorable for the future publication of this work, but before closing this review, I would suggest the authors to include their new Data on TAD conservation as well as on TAD coexpression (graphs provided in the response to reviewer 2). Although it appears to be a negative result, it might definitely be that TAD does not necessary mean co-expression. Instead, co-expression inside TAD is maybe more an exception than a rule.

Of note, a new article in bioRxiv (doi: https://doi.org/10.1101/2021.10.08.463672) is actually going in that direction…

In the field, this will become an important feature into our comprehension of TAD function.

I would also include their graph of contact coexpression conservation (the stronger the co-expression is, the higher the conservation is). This result is expected but very nice to see.

**Authors Response**

**Point-by-point responses to the reviewers’ comments:**

Reviewer #1: Overall, you have done a good job of responding to my previous reviews.

I was, however, confused by this sentence in the revision: "Although cis meta-Hi-C network does not perform better than all individual network, unlike in trans, we still hypothesized that cis meta-Hi-C network have additional coverage for distant gene pairs." Specifically, I can't figure out what you mean by "perform better" here: perform better at what task? Also, the hypothesis is not clear to me: "cis meta-Hi-C network have additional coverage for distant gene pairs." Additional coverage relative to what? I think you mean that co-expressed genes have higher coverage in the meta-Hi-C network than non-co-expressed genes. The text needs to be clarified here.

*Reply: Thank you for pointing it out. For clarification we have updated the text in the main manuscript*

*“Hi-C matrix in cis has high density of contacts at bins near the diagonal and the contact density decreases exponentially as the distance between the bins increases so that even Hi-C networks with higher contact density on average will be highly sparse at distant bins. This makes it difficult to capture functional contacts between distant gene pairs from a Hi-C matrix. Hence, we evaluated the contact coexpression of individual Hi-C networks and meta-Hi-C networks at various linear distance thresholds in cis. We find that for long-range contacts (minimum distance between gene pairs > 1 MB) the additional sequencing depth of meta-Hi-C networks when compared to individual Hi-C networks fully converts into additional performance (Figure 2F). ”*

The compartment analyis is clearer now. However, I think this sentence is misleading: "We find that compartment coexpression and subcompartment coexpression performance is a small fraction of contact coexpression, suggesting ..." It looks to me like the AUC decreases from around 0.65 to around 0.60. I would hardly characterize this as a "small fraction." The difference in performance does support the conclusion that other factors are at play, but the text should more accurately reflect the observed results.

*Reply: Thank you for pointing it out. For clarification we have updated the text in the main manuscript*

*In trans, we find that compartment coexpression and subcompartment coexpression performances are lower than contact coexpression performance, suggesting other trans interactions contribute (Figure 2G).*

Reviewer #2: The authors adequately address my critical points.

I am definitely favorable for the future publication of this work, but before closing this review, I would suggest the authors to include their new Data on TAD conservation as well as on TAD coexpression (graphs provided in the response to reviewer 2). Although it appears to be a negative result, it might definitely be that TAD does not necessary mean co-expression. Instead, co-expression inside TAD is maybe more an exception than a rule. Of note, a new article in bioRxiv (doi: https://doi.org/10.1101/2021.10.08.463672) is actually going in that direction… In the field, this will become an important feature into our comprehension of TAD function.

*Reply: Thank you for your suggestion. We have added TAD conservation and TAD coexpression figures in the manuscript along with the following text*

*“We compared TAD coexpression (defined only in cis), compartment coexpression, and subcompartment coexpression with meta-Hi-C contact coexpression at several resolutions. We used two different methods for calling compartments: an older PCA based method Liberman-Aiden et al 2009 (Lieberman-Aiden2009) and a comparatively recent method Calder (Liu2021). In cis, we find that compartment and subcompartment coexpression is comparable to or better than contact coexpression while TAD coexpression is lower than compartment at up to 10KB resolution (Additional file 1: Fig S3). TADs are often considered functional genomic units and genes within the same TADs tend to be coexpressed (Nora2012). However, unlike compartment and contact coexpression, TAD coexpression does not capture long-range interactions (average TAD size is smaller than 1 MB). This likely explains the non-random yet low performance of TAD coexpression (AUC 0.55). We also evaluated the conservation of TADs and boundaries across all individual Hi-C matrices (Additional file 1: Fig S4). The number of TADs conserved across experiments decreases relatively rapidly and we did not find any TAD which was conserved across all the experiments.”*

I would also include their graph of contact coexpression conservation (the stronger the co-expression is, the higher the conservation is). This result is expected but very nice to see.

*Reply: Thank you for your suggestion. We have added contact coexpression conservation figure along with the following text in the main manuscript*

*“As expected, we also find that the strongest coexpressed genes have the most contact coexpression conservation (Additional file 1: Fig S5).”*
